# Effect of different kinematics and perforation diameter on integrated electronic apex locator accuracy in detecting root canal perforations

**DOI:** 10.1111/eos.70020

**Published:** 2025-06-04

**Authors:** Ecenur Tuzcu, Safa Kurnaz

**Affiliations:** ^1^ Department of Endodontics Faculty of Dentistry Kutahya Health Sciences University Kutahya Türkiye

**Keywords:** electronic apex locator, reciprocating, root canal preparation, root perforation, rotary

## Abstract

This study aimed to evaluate the precision and reliability of the integrated electronic apex locator in determining various root perforation diameters and to examine the impact of instrumentation kinematics on perforation site detection. One hundred extracted single‐root mandibular premolars were artificially perforated at five distinct diameters (0.25–1.25 mm), located 5 mm above the root apex. The teeth were then divided into 10 groups based on perforation size and kinematic subgroup (rotary, reciprocation; *n *= 10/subgroup). Actual distances to the perforation site (actual length) were measured under a dental operating microscope. Electronic distances (electronic length) were determined using an endodontic motor with an integrated electronic apex locator using rotary or reciprocating instrumentation kinematics during root canal treatment. To quantify the actual length to the electronic length discrepancies, individual tooth measurements were compared. The integrated electronic apex locator failed to identify perforations ≤ 0.50 mm in diameter. Electronic length measurements were similarly accurate for 1.00 and 1.25 mm perforations but more precise than for 0.75 mm perforations. Instrumentation kinematics did not affect the electronic length measurement accuracy. Integrated electronic apex locators failed to detect small perforations during root canal treatment. The perforation diameter influenced the detection accuracy of integrated electronic apex locators, whereas kinematics had no effect.

## INTRODUCTION

A root perforation is an abnormal communication between the root canal system and the surrounding periodontal tissues. While commonly induced iatrogenically during dental procedures, root perforations can also originate from resorptive processes [1, 2]. These defects compromise the tooth's structural integrity, rendering it susceptible to infection and potential loss. Prompt identification and appropriate management of root perforations are essential for preserving the tooth, as the prognosis is contingent upon factors such as perforation dimensions, location, and duration [1, 3].

Accurate localization of a root perforation is crucial in root canal treatment to prevent the extrusion of irritants into periradicular tissues and to avoid instrument overextension [4]. A variety of diagnostic techniques, including direct observation of bleeding, indirect evaluation of bleeding with a paper point, radiographic evaluation, cone‐beam computed tomography (CBCT), and electronic apex locators, are used for root perforation detection [1]. Although conventional radiography is essential, its two‐dimensional nature often limits its usefulness in identifying root perforations on the buccal or lingual root aspects [5].

CBCT provides a three‐dimensional view, which may enhance diagnostic accuracy in the detection of root perforations. Although image quality can be affected by factors such as voxel size and artifacts from patient movement or radiopaque materials [6, 7], recent advancements in post‐processing software have significantly improved image clarity by reducing blooming artifacts and noise [8, 9, 10, 11]. Additionally, radiological protection principles, including “As Low As Reasonably Achievable” (ALARA), continue to regulate CBCT use, ensuring that radiation exposure is kept to the minimum necessary for clinical applications, while emerging radiation protection strategies further reduce associated risks [12]. Despite the advancements in post‐processing software, CBCT still has some limitations, such as high cost, limited availability, and the need for specialized interpretation [13]. Alternatively, the electronic apex locator offers a cost‐effective, minimally invasive, and reliable method for detecting root perforations, and remains an indispensable tool in clinical endodontics for accurate root perforation detection [14, 15].

Electronic apex locators are commercially available as single units or integrated components of endodontic motors. Integrated electronic apex locators enable clinicians to shape and clean root canals while continuously monitoring the file's position within the canal [16, 17]. Specific endodontic motors are nowadays often equipped with an integrated electronic apex locator. Controlled by a foot pedal, they offer adjustable speed and torque settings, and they are compatible with reciprocating and continuous rotary nickel‐titanium systems. Such devices allow for simultaneous length control or independent length measurement [18].

Nickel‐titanium file systems have become increasingly popular, with a wide variety of systems available that operate using either rotational or reciprocating movements. These systems differ in the directions they move during root canal shaping and by the mechanisms they use. Various studies have investigated the kinematics in relation to their effect on factors such as cleaning and shaping efficiency, apical extrusion, fracture resistance, and the reduction of intracanal bacteria [19, 20, 21, 22]. The movement types may affect the accuracy of electronic apex locators, depending on their motion characteristics and the debris generated during preparation. While some studies indicate that kinematic movements can influence the performance of apex locators [16], data, particularly regarding the effects on perforation detection, are limited. Although research on integrated electronic apex locators compared with single units is relatively scarce, it has been suggested that integrated systems can precisely measure the root canal working length [23, 24, 25, 26]. Several studies have focused on the accuracy of different apex locators in detecting root perforations [14, 15, 27–29], but there are still limited data on the detection of minimal perforation sizes [30]. Furthermore, there is a lack of research examining the performance of integrated electronic apex locators in detecting perforations during root canal treatment with different kinematic movements. This investigation seeks to evaluate the influence of differing perforation sizes and nickel‐titanium instrument kinematics on the efficacy of an endodontic motor with an integrated electronic apex locator in pinpointing simulated root perforations.

The null hypotheses tested in this study are as follows: first, that different perforation diameters do not affect the accuracy of detecting root perforation locations using an integrated electronic apex locator; and second, that the rotary and reciprocating movements used in root canal treatment do not affect the accuracy of detecting root perforation locations using an integrated electronic apex locator.

## MATERIAL AND METHODS

This in vitro study was approved by the local university ethics committee (reference number: 2023/06–28). The g*power program ver. 3.1.9.7 (https://www.psychologie.hhu.de/arbeitsgruppen/allgemeine‐psychologie‐und‐arbeitspsychologie/gpower) was used to determine the sample size. A minimum of eight samples per group was required for the 5 × 2 one‐way analysis of variance design, with 80% power, a 0.5 effect size (Cohen's f), and a 0.05 alpha value [16, 30]. Due to potential experimental errors, the appropriate number of samples for each group was determined to be 10, and the calculated power value was found to be 94%.

A detailed examination of possibly eligible human teeth was conducted under a dental operating microscope (OMS 2350, Zumax). Periapical radiographs were captured in both the buccolingual and mesiodistal planes to assess each tooth. The aim of these procedures was to detect and exclude teeth with caries, cracks, fractures, calcification, internal root resorption, or previous root canal treatment, and to confirm the existence of a single root canal. In total, 150 mandibular premolar teeth meeting the selection criteria with approximately the same dimensions were chosen from specimens extracted for orthodontic or periodontal reasons at the Department of Oral and Maxillofacial Surgery, Kütahya Health Sciences University, with patient consent. Soft tissue residues and calculus were scraped from the root surfaces with periodontal curettes, and the teeth were subsequently kept in a 0.1% thymol solution (Norateks Kimya) at 4°C for storage until required. To access the root canal, the crown of each specimen was removed using a diamond disk at the cementoenamel junction, establishing a standardized measurement point at 18 mm [26, 31]. A suitable barbed broach (Dentsply Maillefer) was employed for canal debridement. Canal negotiation and apical foramen patency were manually established using a stainless steel #10 K‐file (Dentsply Maillefer) through gentle clockwise and counterclockwise rotations with apical increments of 1–2 mm. Apical preparation was achieved using up to a #15 K‐file [30]. Subsequent dental operating microscope examination of the roots was conducted using a #15 K‐file inserted until its tip was visualized within the apical foramen. Teeth in which the #15 K‐file extended beyond the apex with a visible tip, yet a #20 K‐file encountered resistance, were included in the study. Five milliliters of 2.5% sodium hypochlorite (Wizard, Rehber Kimya) and 5 mL of saline solution (0.9% NaCl; Polifarma Ilaç) were utilized by the operator for each tooth during canal exploration. Teeth that did not meet the study criteria were replaced with suitable specimens. From the initial 150 teeth collected, 100 that fully met the criteria were included in the study. The included teeth were individually numbered and randomly assigned to one of five main groups based on perforation diameter, using a computer‐generated random sequence (www.random.org). Each main group was then further subdivided into two subgroups based on kinematics (reciprocating and rotary), using the same random sequence method. To ensure that the specimens were of similar size, the mesiodistal and buccolingual root diameters at the cementoenamel junction and at a point 5 mm from the apex (the intended perforation site) were recorded using digital calipers (Hogetex).

External root surfaces were delineated with an acetate pen to pinpoint the perforation site, which was positioned 5 mm apical to the root apex [14, 30]. Diamond bur diameters were verified using digital calipers. Artificial root perforations were subsequently created at the marked locations on the external lateral root surfaces in the distal region of the tooth using diamond burs directed perpendicularly to the root's long axis under water coolant. To minimize perforation size variations due to bur attrition, a new bur was employed for every five teeth. Root perforation diameters were established at 0.25 mm for Group 1, 0.50 mm for Group 2, 0.75 mm for Group 3, 1.00 mm for Group 4, and 1.25 mm for Group 5 [30]. The teeth were then divided into two subgroups for each root perforation diameter: subgroup “a” for teeth that would use rotation kinematics and subgroup “b” for teeth that would use reciprocation kinematics.

Actual lengths were measured from the cementoenamel junction to the perforation site's coronal margin. Actual length calculations were based on distances from the rubber stop to the tip of a #20 K‐file, visualized at the root perforation area under a dental operating microscope at 19.4x magnification.

Plastic containers were filled with freshly mixed alginate. For measurements, the Gold Reciproc motor (VDW) was used, in the following named as the endodontic motor. Root samples extending to the cementoenamel junction and the labial clip of the integrated electronic apex locator of the endodontic motor were subsequently embedded within the alginate (Tropicalgin, Zhermack). Following alginate solidification, the teeth were retrieved, and alginate remnants within the perforation site were eliminated using a probe and air–water spray. The teeth were then reinserted into their sockets for electronic length measurement [16, 30]. To maintain adequate alginate moisture, all measurements were conducted within 30 min of the alginate mold creation.

The endodontic motor was calibrated, and its integrated electronic apex locator function and “auto‐apex‐stop” function were activated. Subsequently, 1 mL of 2.5% sodium hypochlorite was irrigated into the root canals of the alginate‐embedded teeth, with excess solution removed from the coronal region using a sponge.

For the rotary groups (groups 1a–5a), the electronic lengths were established during root canal treatment using 35.04 One Curve (Micro‐Mega) files. These files were employed with a gentle in‐and‐out motion, a continuous rotary speed of 350 rpm, and 2.5 Ncm of torque, as per the manufacturer's guidelines. The file was retracted after every 2–3 mm of in‐and‐out motion, debris was eliminated from the flutes using a sponge, and the root canals were irrigated with 2.5 mL of 2.5% sodium hypochlorite using a 30G (Endo‐Top, Cerkamed) side‐perforated irrigation needle. Recapitulation was performed with a #15 K‐type file. Excess solution was removed from the coronal region using a sponge before file reinsertion.

In the reciprocation groups (groups 1b–5b), electronic lengths were determined using 35.04 One Reci (Micro‐Mega) files during root canal treatment with reciprocating motion. The endodontic motor was configured for reciprocation mode for One Reci file use. The file was employed with slow in‐and‐out pecking motions within 2–3 mm amplitudes using light pressure, following the manufacturer's recommendations. After every three pecking motions, the file was removed, debris was cleaned from the flutes, and the root canals were irrigated with 2.5 mL of 2.5% sodium hypochlorite via a 30G side‐perforated irrigation needle. Recapitulation was performed with a #15 K‐type file.

Electronic length measurements of the distance to the perforation site and the impact of nickel‐titanium instrument kinematics on measurement accuracy and variability were assessed using an integrated electronic apex locator within the endodontic motor's “auto‐apex‐stop” function for each tooth [16]. A new instrument with a tight rubber stopper was employed for each tooth during root canal treatment to facilitate visualization of the correct measurement indicated by the integrated electronic apex locator. The instrument was automatically stopped when it reached the perforation site. Subsequently, the operator adjusted the rubber stopper to the tooth's reference point, removed the instrument from the canal, and measured the distance provided by the integrated electronic apex locator using digital calipers. The electronic length measurement for each tooth was recorded independently. All measurements were conducted by the same operator, who was experienced in utilizing the integrated electronic apex locator of the endodontic motor. All measurements were performed using a digital caliper with 0.01 mm accuracy under the dental operating microscope.

Calculations were performed by subtracting actual lengths from electronic lengths to determine differences. Positive values (+) denoted a longer electronic measurement than the actual length, while negative values (−) indicated a shorter electronic measurement than the actual length. A value of 0 represented a matching measurement. Clinically acceptable electronic length measurements were ± 0.5 mm of the actual lengths [14, 15].

Statistical analyses were performed using the SPSS (IBM SPSS for Windows, version 26) statistical package. Data normality was assessed using the Shapiro‐Wilk test. One‐way analysis of variance and independent *t*‐tests were employed for statistical comparisons. The Tukey test was subsequently applied for post hoc group comparisons. Statistical significance was set at *p* < 0.05.

## RESULTS

The results of the Shapiro‐Wilk test confirmed the normal distribution of the data. Statistical analysis revealed no significant differences in the mesiodistal and buccolingual root diameters at both the cementoenamel junction (*p* = 0.976 and *p* = 0.334, respectively) and 5 mm from the apex (the site where the perforation was created; *p* = 0.140 and *p* = 0.116, respectively), indicating dimensional homogeneity among teeth in the groups.

**FIGURE 1:**
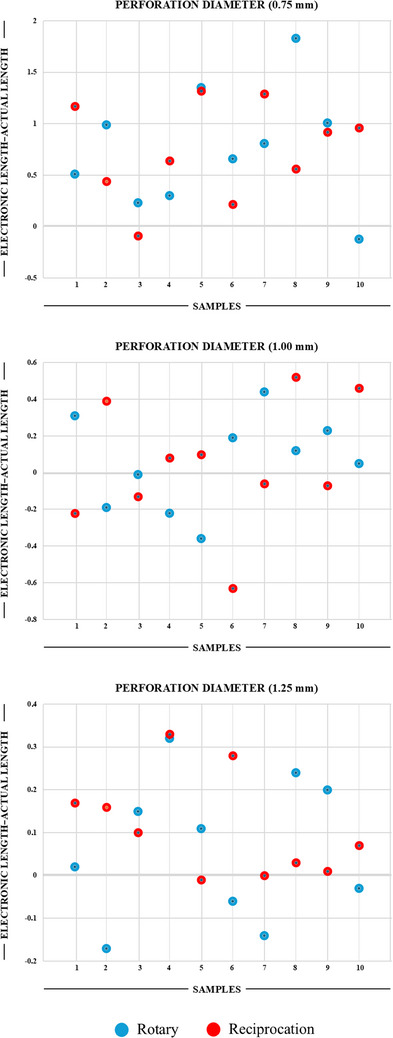
Distribution of differences between the electronic length and the actual length measurements according to the diameter of the perforations made in the teeth in each group.

Mean differences between the electronic lengths and the actual lengths, accompanied by standard deviation values for each perforation diameter, are presented in Table 1. The distribution of differences between the electronic lengths and the actual lengths for various reference intervals is detailed in Table 2 and Figure 1.

**TABLE 1:** Mean value, SD, and confidence intervals of the difference between the electronic length and the actual length for different perforation sizes and instrumentation kinematics.

	Perforation diameter	Overall accuracy		Rotary		Reciprocation	
		Mean ± SD	95% CI	Mean ± SD	95% CI	Mean ± SD	95% CI
**Difference**							
Electronic −	0.75 mm	0.77 ± 0.48^a^	0.55–0.99	0.78 ± 0.54	0.40–1.16	0.76 ± 0.44	0.45–1.07
Actual length (mm)	1.00 mm	0.24 ± 0.18^b^	0.16–0.32	0.21 ± 0.13	0.12–0.31	0.27 ± 0.22	0.11–0.42
	1.25 mm	0.13 ± 0.11^b^	0.08–0.18	0.14 ± 0.10	0.08–0.21	0.12 ± 0.12	0.03–0.20

*Note*: Values in the same column with different superscript letters are statistically different (*p* < 0.05). Specifically, the 0.75 mm perforation group (a) is significantly different from the 1.00 mm and 1.25 mm groups (b), whereas there is no significant difference between the 1.00 mm and 1.25 mm groups.

Abbreviation: SD, standard deviation.

**TABLE 2:** Distribution of the electronic length—actual length differences across groups defined by varying perforation diameters and filing technique.

	Perforation diameter					
	0.75 mm		1.00 mm		1.25 mm	
Difference	Rotary	Reciprocation	Rotary	Reciprocation	Rotary	Reciprocation
Electronic − Actual length (mm)	%	%	%	%	%	%
< −0.51	0	0	0	10	0	0
−0.5 to −0.01	10	10	40	40	40	10
0.00	0	0	0	0	0	10
0.01 to 0.50	20	20	60	40	60	80
> 0.51	70	70	0	10	0	0

*Note*: Negative values represent a file position coronal to the perforation, while positive values indicate an apical file position.

In the present study, electronic lengths could not be determined for all samples with perforation diameters 0.25 and 0.50 mm using an endodontic motor with an integrated electronic apex locator. Electronic length—actual length values exhibited similarity between teeth with perforation diameters of 1.00 and 1.25 mm, with no statistically significant difference observed (*p* = 0.487). Conversely, teeth with a perforation diameter of 0.75 mm demonstrated significantly higher electronic length—actual length values than both teeth with 1.00 and 1.25 mm perforation diameters (*p* < 0.0001). Across all groups, regardless of the perforation diameter, the type of kinematics used (rotation or reciprocation) did not influence the perforation site measurements obtained using an endodontic motor with an integrated electronic apex locator as evidenced by no statistically significant differences.

## DISCUSSION

The present investigation sought to assess the influence of varying perforation diameters and different kinematics on root perforation localization. Five distinct perforation diameters (0.25, 0.50, 0.75, 1, and 1.25 mm) and two kinematic modalities (rotary and reciprocating) were examined in an in vitro setting utilizing an endodontic motor equipped with an integrated electronic apex locator. The results indicated that root perforations with a diameter of 0.5 mm or less were undetectable by the integrated electronic apex locator. In contrast, root perforations of 0.75 mm and greater exhibited clinically acceptable localization accuracy. A positive correlation was observed between the perforation size and the precision of location determination. No discernible disparity in perforation localization was identified between rotary and reciprocating motion. Consequently, the perforation diameter emerged as a critical determinant of accurate root perforation location, whereas kinematic variation exerted no significant impact. Based on these findings, the first hypothesis was rejected, while the secondary hypothesis was accepted.

The outcome and prognosis for teeth affected by root perforations are contingent upon several factors, including the perforation site, dimensions, temporal interval between occurrence and treatment initiation, precise localization, and the capacity to effectively seal the perforation via endodontic procedures [1]. Accurate diagnosis of root perforations situated on the lingual or buccal root surfaces can pose significant challenges, even for practitioners with substantial endodontic experience, owing to the superimposition of anatomical structures inherent in two‐dimensional conventional radiographic imaging [32]. Radiographic detection of root perforations is limited by its susceptibility to inaccuracies introduced by anatomical structures and its two‐dimensional representation [33]. Despite the three‐dimensional imaging capabilities of CBCT, research conducted by Shemesh et al. [7] has demonstrated that even this technique may be inadequate for identifying strip perforations in root‐filled teeth. The development of a precise device for locating root perforations is indispensable for achieving successful treatment outcomes. In vitro investigations evaluating the accuracy of electronic apex locators in detecting root perforations have yielded results supporting their clinical suitability for this purpose [4, 14, 15, 27].

All controllable variables were standardized in this investigation. The perforation size and instrumentation kinematics were designated as study parameters. Extracted mandibular premolars were used, and to establish a uniform reference plane for tooth crowns and to ensure consistency, the root length was decoronated at 18 mm [26, 31]. Given the influence of root canal dimensions on the electronic apex locator accuracy, teeth with comparable dimensions were selected, and care was taken to ensure that the file size was uniform [34]. To ensure standardization, a #20 file was selected as the initial apical file. Since adequate canal enlargement up to three file sizes beyond the initial apical file has been shown to be sufficient, #35 was selected as the master apical file [35]. The root canals were prepared using single‐file systems with a #35 apical diameter, one designed for rotary motion and the other for reciprocating motion, both of which have been shown to be effective in root canal treatment according to previous studies [36, 37]. In addition, all measurements were made with a digital caliper under a dental operating microscope to increase the accuracy of the measurements.

Previous investigations have employed a variety of electroconductive substances, including alginate, agar‐agar, saline, and gelatin, for in vitro assessments of electronic apex locators [14, 29, 38, 39]. Among these, the alginate model has consistently demonstrated superior reliability in evaluating the accuracy of electronic apex locator measurements, as confirmed by many studies [40, 41]. Research by Duran‐Sindreu et al. revealed no statistically significant difference between the in vivo and in vitro alginate model findings [42]. Given its ease of handling, effective conductivity, high elasticity, and viscosity, alginate was selected as the embedding material for this study. These properties facilitate a precise and adaptable fit around the root surface, effectively simulating periodontal tissue conditions [43].

Previous investigations into root perforations have employed varying perforation dimensions. Fuss et al. [4] induced 0.27 mm root perforations on the external root surfaces of teeth, demonstrating the clinical acceptability of electronic apex locator devices for this root perforation size. Kaufman et al. [44] introduced larger (0.55–0.60 mm) and smaller (0.25 and 0.40 mm) root perforations in the middle third of the root, revealing no significant disparity between perforation sizes but highlighting the superior accuracy of Root ZX compared with other electronic apex locators. Our study utilizing the Gold Reciproc motor was unable to detect root perforations of 0.25 and 0.50 mm, contrasting with the study findings of Fuss et al. [4] and Kaufman et al. [44]. These discrepancies can be attributed to different experimental protocols, variations in tooth morphology, and dissimilarities in root perforation creation techniques. Unlike the aforementioned studies, which relied solely on electronic apex locators, our research focused on an electronic apex locator integrated within the tested endodontic motor during root canal treatment while employing both rotary and reciprocation file kinematics. Additionally, while prior research simulated strip perforations, our focus was on mimicking external root resorption through perpendicularly oriented diamond bur perforations on the external root surface. To control for dentin thickness variability, we standardized our sample to similarly sized teeth and exclusively utilized distal root surfaces for perforation creation [29].

Aydın and Bulut [45] investigated the influence of root perforation diameter on the efficacy of two electronic apex locators (Dentaport ZX and Gold Reciproc motor) by employing perforation sizes of 0.4 and 1.00 mm. Their findings indicated that the Dentaport ZX exhibited superior reliability in detecting 0.4 mm perforations compared with the Gold Reciproc motor, and that no statistically significant differences in locating the perforated area were observed between the two devices for the 1.00 mm perforation group. These results align with our findings, demonstrating the enhanced ability of the Gold Reciproc motor to identify perforation locations with increasing diameter. Our study expanded upon these findings by examining a wider range of perforation diameters (0.25, 0.50, 0.75, 1.00, and 1.25 mm), consistent with the methodology of Koç et al. [30]. In their study, they employed six electronic apex locators and five different perforation sizes to assess their impact on root perforation detection. Our results corroborate the findings of Koç et al. [30], indicating an inability to detect root perforations of 0.25 and 0.50 mm using electronic apex locators. Furthermore, both studies demonstrated a positive correlation between perforation diameter and the accuracy of locating the root perforation.

The advent of electronic apex locators has significantly enhanced the precision of root canal length determinations [46]. These devices exploit the human body to establish an electrical circuit [47]. One electrode interfaces with the endodontic file within the root canal, while the other contacts the patient's lip. Upon reaching the periodontal tissues, the file completes the circuit [48]. Modern electronic apex locators function by employing diverse frequencies to calculate the ratio between distinct electrical potentials, which correlate with impedance. The presence of electrolytes does not necessitate canal drying prior to electronic apex locator utilization [49]. The efficacy of electronic apex locators in wet canal environments has been a subject of investigation. Irrigation solutions are vital for cleaning and thoroughly disinfecting the canal during root canal treatment [50]. Various studies have assessed the accuracy of these devices in dry conditions or in conjunction with different irrigating agents [15, 27, 28]. Sodium hypochlorite, renowned for its bactericidal and tissue‐dissolving properties, is a primary irrigant in root canal treatment [50]. Its electroconductive nature, however, raises concerns regarding potential interference with electrical resistance and impedance measurements [51]. Nevertheless, Diemer et al. [51] demonstrated that varying sodium hypochlorite concentrations exerted no detrimental influence on electronic apex locator reliability. These findings underscore the clinical accuracy of electronic length measurements in the presence of diverse sodium hypochlorite concentrations, with minimal impact on electrical readings at low current levels. In another study, it was shown that since the simulated root perforation was made to the proximal root plane, well‐flowing liquid types are more advantageous in reaching the external proximal root surface; therefore, liquid irrigants resulted in higher accuracy compared with gel‐type irrigants for locating root perforations [29]. Consequently, a 2.5% sodium hypochlorite solution, which is commonly used during root canal treatment, was selected for this investigation to better reflect clinical practice. In this study, the inability to detect electronic length measurements in smaller (0.25 and 0.50 mm) perforation areas may be attributed to inadequate solution penetration and insufficient electroconductivity. As the perforation size increased, enhanced electroconductivity may have facilitated more precise measurements. Nevertheless, further investigation is required to establish the smallest perforation size at which various solutions can facilitate adequate electroconductivity for precise measurements. Subsequent research endeavors may be designed to address this knowledge gap.

de Almeida Gardelin et al. [16] utilized the same endodontic motor as in the present study to examine the impact of nickel‐titanium instrument kinematics on root canal working length accuracy as determined by an integrated electronic apex locator during the glide path and end of shaping stages. Their findings indicated no discrepancy in the glide path stage but revealed enhanced accuracy with rotary kinematics in the end of shaping stage. Conversely, the present investigation did not identify a difference between the two kinematic modes. These conflicting results may be attributed to variations in tooth selection and the distinct outcome measures, as working length determination was the focus of de Almeida Gardelin et al.’s [16] research, whereas our study centered on perforation detection.

This in vitro study provides valuable insights into the performance of electronic apex locators in detecting root perforations but has several limitations. First, the artificially induced perforations, while necessary for standardization, may not fully replicate clinical conditions, where factors such as tissue damage, inflammation, and infection influence electrical conductivity. The absence of living tissue, electroconductive fluids (e.g., blood and saliva), and the periodontal ligament's distinct resistance properties limit the direct clinical applicability of our findings. Future studies should incorporate more biologically relevant models to better simulate in vivo conditions. Additionally, our results are specific to the selected tooth groups and electronic apex locator models, limiting their generalizability. Variations in root anatomy, dentin thickness, and device technology could influence accuracy. Moreover, factors such as tooth condition (e.g., caries, restorations, prior treatments) and operator experience were not considered, though they likely impact diagnostic precision. Furthermore, this study focused on standardized perforation induction, which may not reflect the full range of clinical perforation types. Investigating diverse perforation morphologies, including those from iatrogenic errors or resorption, as well as the effects of different instrumentation techniques, would provide a more comprehensive understanding. Future research should address these variables to refine clinical protocols and to enhance diagnostic accuracy in endodontics.

In conclusion, this study demonstrates that electronic apex locator accuracy is influenced by perforation diameter. Integrated electronic apex locators reliably detect perforations of 1.00 mm or larger, while supplementary methods may be necessary for smaller perforations (≤0.50 mm). Additionally, no significant difference was observed between rotary and reciprocation kinematics in perforation detection, supporting the clinical applicability of both kinematic modes with integrated electronic apex locators.

## AUTHOR CONTRIBUTIONS

**Conceptualization**: Ecenur Tuzcu, Safa Kurnaz. **Methodology**: Ecenur Tuzcu, Safa Kurnaz**. Investigation**: Ecenur Tuzcu, Safa Kurnaz. **Resources**: Ecenur Tuzcu, Safa Kurnaz. **Data curation**: Ecenur Tuzcu. **Writing—original draft preparation**: Ecenur Tuzcu. **Writing—review and editing**: Safa Kurnaz. **Visualization**: Ecenur Tuzcu, Safa Kurnaz. **Project administration**: Ecenur Tuzcu, Safa Kurnaz.

## CONFLICT OF INTEREST STATEMENT

The authors declare no conflicts of interest.

## Data Availability

The datasets used and/or analyzed during the current study are available from the corresponding author on reasonable request.
